# An artificial intelligence based news feature mining system based on the Internet of Things and multi-sensor fusion

**DOI:** 10.7717/peerj-cs.1428

**Published:** 2023-06-21

**Authors:** Zhuozheng Xie, Junren Wang

**Affiliations:** Yiwu Industrial and Commercial College, Yiwu, China

**Keywords:** IoT technology, News communication, Sensors, Artificial intelligence

## Abstract

The application of Internet of Things (IoT) technology in news media communication has significantly enhanced the effectiveness and coverage of news data releases. However, as the scale of news data continues to grow, traditional IoT approaches face challenges such as slow data processing speed and low mining efficiency. To address these issues, a novel news feature mining system combining IoT and Artificial Intelligence (AI) has been developed. The hardware components of the system include a data collector, a data analyzer, a central controller, and sensors. The GJ-HD data collector is utilized to gather news data. Multiple network interfaces are designed at the device terminal to ensure data extraction from the internal disk in case of device failure. The central controller integrates the MP/MC and DCNF interfaces for seamless information interconnection. In the software aspect of the system, the network transmission protocol of the AI algorithm is embedded, and a communication feature model is constructed. This enables fast and accurate mining of news data communication features. Experimental results demonstrate that the system achieves a mining accuracy of over 98%, enabling efficient processing of news data. Overall, the proposed IoT and AI-based news feature mining system overcomes the limitations of traditional approaches, allowing for efficient and accurate processing of news data in a rapidly expanding digital landscape.

## Introduction

The rapid development of information technology provides a good transmission condition for news communication. In a narrow sense, the news is defined as a style that describes real-time events through brief language. News data dissemination aims to make certain events at home and abroad known to the public (Franceschi, Pareschi & Zanella, 2022). The efficiency of news communication is related to its channels and characteristics. With the progress of social science and technology, the way, mode, carrier and presentation of news communication have changed (Li, 2022). The characteristics of news communication are mainly divided into three parts: news expression, narrative style and big data state, which contain many dimensions and complex data types (Alotaibi & Alhammad, 2022). Therefore, realizing the effective and accurate processing and analysis of news data and enhancing the effectiveness of news communication is a problem worth studying.

In the early days, related researchers used data modeling, computer simulation and other technologies to process news data. For example, Park, Park & Hu (2021) modeled the theme and meaning of tourism news and realized the tourism demand forecast. Qi, Yang & Wang (2019) analyzed news data through computational visualization technology to help journalists find the inherent logic in news data. Yang & Jin (2020) manually classified the collected news data and used computer simulation technology to explore the propagation characteristics of these news data. Although the above methods have improved the dissemination efficiency and coverage of news data to a certain extent, the rapid development of the Internet has made the scale of news data more extensive. These methods have been unable to achieve efficient news data analysis and need further improvement (Rath, Salecha & Srivastava, 2022). The IoT connects objects based on the Internet (Ammi, Alarabi & Benkhelifa, 2021), which realizes the intelligent management of information exchange by connecting objects with the Internet. The application of IoT mainly includes RFID technology (Charléty et al., 2022), sensor technology (Huang & Lei, 2022) and embedded system technology (Mou, 2022). Among them, sensor technology can improve the data processing speed, enhance the network capacity and speed, and relieve the pressure of large-scale news data processing to a certain extent.

The unique characteristics of news data require specific data transmission capabilities that are not effectively addressed solely by IoT technology. Relying solely on IoT for news data processing limits the transmission effectiveness and overall impact of news data. To overcome this limitation, researchers have turned to AI algorithms to match and predict the features of news data, yielding promising results in data analysis. In the earlier stages of research, supervised learning techniques were predominantly used to extract distinct news features from text content, user information, and communication modes. Traditional machine learning models were commonly employed for this purpose (Wu, Song & Zhu, 2015). However, with the emergence of deep learning models, methods based on neural networks have demonstrated improved feature extraction capabilities and have achieved notable advancements in extracting features from news data. The utilization of deep learning models has allowed for more sophisticated and effective feature extraction, enhancing the analysis of news data. This shift in approach has opened up new possibilities for understanding and leveraging the unique characteristics of news data through AI-based methods. Ma, Gao & Wong (2018) constructed a new data transmission protocol based on a recurrent neural network, which captures the semantic differences between news sources and their forwarding in Weibo to predict the next transmission point according to the semantic changes. Khattar et al. (2019) used recurrent neural networks based on trees to capture the potential semantic information features of news in the communication structure. Liu & Wu (2018) integrated the network communication protocol into the variational automatic encoder, which is used to obtain the text features and image features covered by a post to determine whether the post is false news or not. Wang et al. (2022) used a neural network to capture the changes in user characteristics of relevant news participants along the propagation path. The above methods only consider individual news, ignoring the structural correlation between social network information. They can be connected if the same user publishes or forwards multiple news. Such associations can share data between connected instances and help each other detect and improve news data processing performance. For example, Huang et al. (2020) used neural networks to express news articles, creators and themes in a fusion way and to mine the structural features of news data. Castillo, Mendoza & Poblete (2011) adopted a neural network to capture semantic information of news data from posts, comments and heterogeneous graphs constructed by related users on social networks. Altheneyan & Alhadlaq (2023) employed machine learning and Spark technology to recognize the fake news and achieve a superior classification performance comparing with other competitive methods. Hamborg et al. (2017) proposed the crawler and extractor to help researcher obtain the news information online and to summary the major elements of news. Ghayoomi (2023) aimed at filter unnecessary information in news dissemination to propose the XLM-RoBERTa representation by CNN, which can spot English and Persian fake news. However, these methods emphasize the semantic changes of text data in the process of news communication, ignoring the dissemination characteristics of news data, and can’t achieve better performance.

This article aims to achieve precise mining and processing of news data while enhancing the efficiency of news communication. To accomplish this, the article introduces a network transmission protocol embedded within an AI algorithm based on IoT principles, and designs a news data mining system. The contributions of this work are outlined as follows:

Hardware design: The system incorporates high-performance components such as a data collector, data analyzer, central controller, and sensors. These hardware elements have been optimized to facilitate seamless function calls within the system software domain.

Network operation protocol: Considering the dissemination of news data and the requirements for efficient mining, a network operation protocol based on an AI algorithm has been developed and integrated into the system. This protocol enhances the transmission and processing of news data, enabling accurate and efficient mining of valuable information.

Through these contributions, the proposed system offers a comprehensive solution for effective news data mining and processing. By leveraging IoT principles, AI algorithms, and a carefully designed network operation protocol, the system aims to optimize the communication and analysis of news data, thereby improving the overall efficiency of news dissemination.

## Hardware Design

**Figure 1:**
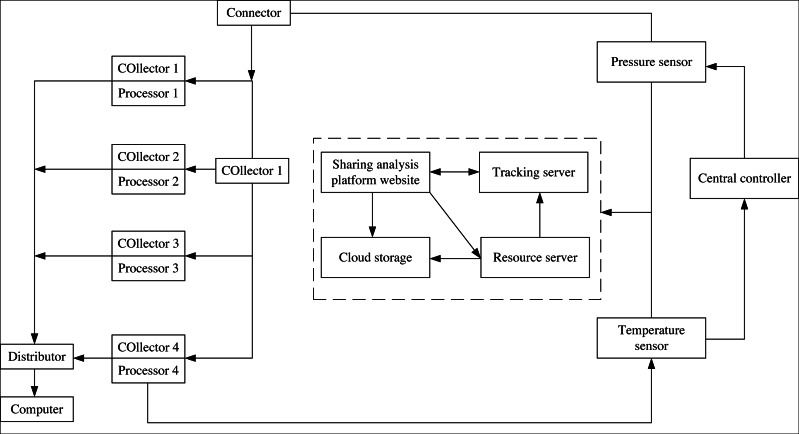
Hardware structure of news data mining system.

The hardware design relies on the Internet, and IoT technology processes news data. The overall architecture of the system is shown in Fig. 1.

### Data collector

**Figure 2:**
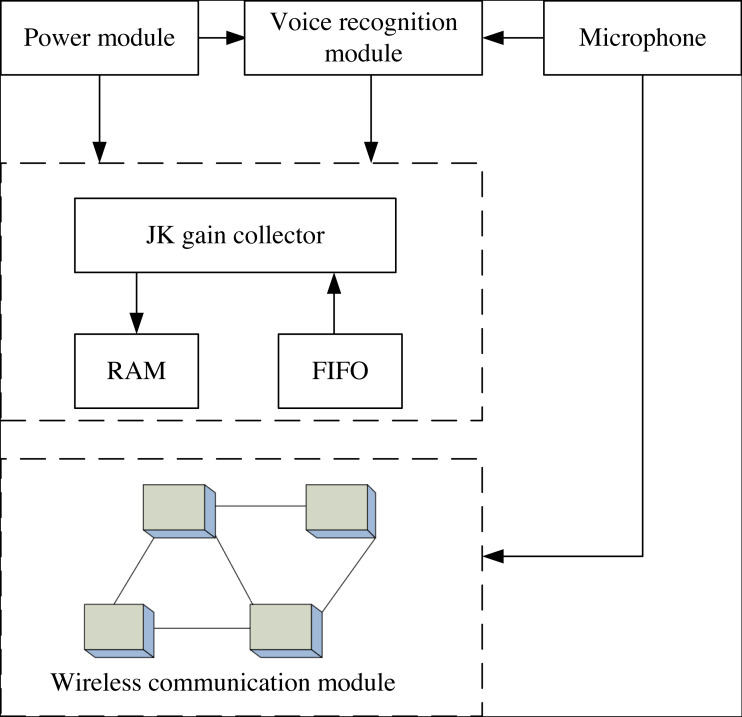
Structure of GJ-HD data collector.

The authenticity of data is the basis for the extraction of news big data propagation characteristics and the success of data mining. The hardware regional data collector can ensure the integrity of news data on the one hand and verify the authenticity of news data on the other hand. Therefore, the GJ-HD data collector (Wang et al., 2019; Roh, Heo & Whang, 2019) is adopted in this article, where the channel type of data information transmission in this data collector is single-ended 16 channels. Working frequency is different for different types of text news data, and the maximum amplitude can reach 40M. To improve the adaptability of the data collector, two types of data buffer disks, RAM (Chang et al., 2016) and FIFO (Cummings, 2002), are used, which have high speed and confidentiality. The structure of the GJ-HD data collector is shown in Fig. 2.

 GJ-HD data collector has a working voltage of 220 V, a built-in battery capacity of 5,000 mV, and a data resolution of 12 bits. GK gain card is adopted to realize all the conversion of news information. The actual memory of the data collector is 64 GB, and a virtual hard disk of 200 GB is designed. The data acquisition rate verified in the system is 640 kb/s.

### Data analyser

**Figure 3:**
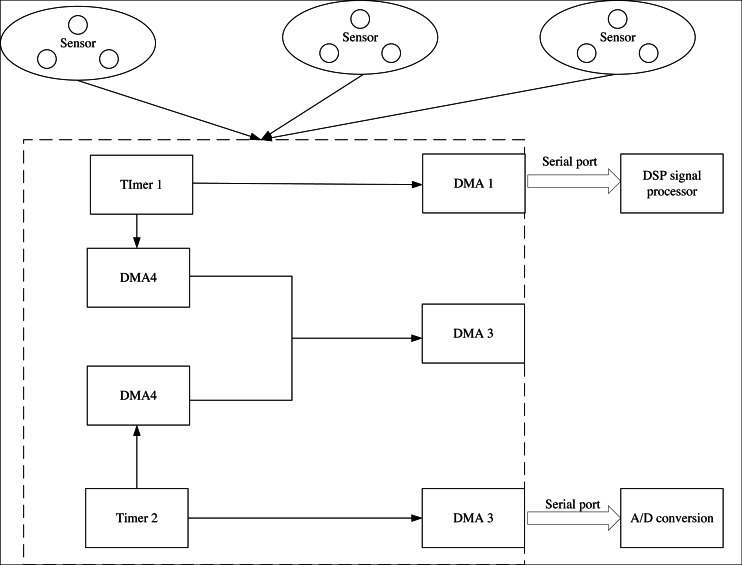
Structure of data analyzer.

The core of this system design is to complete the extraction and mining of news data dissemination characteristics, and the data analyzer is essential. The task of this device is to analyze all the data in the big news data packet and analyze the core ideas, values, keywords and other information points of big news data to lay a data foundation for mining news data dissemination characteristics. According to the work to be done by the above data analyzer, this article selects the i9 series data analyzer, which has a running space of 128 GB. According to the running requirements, it also has four external interfaces for mobile space. The structure of the data analyzer is shown in Fig. 3.

From Fig. 3, the analysis rate of the device can reach 650KB/s, and it adopts a USB 3.0 interface protocol to stabilize the output stability of data. The LAN channel number of the device is 1, and the CAN channel number is 2, which supports candf operation technology. The output rate of the instrument for big data texts is 10,000 frames per second, which ensures the working efficiency of the instrument. The working voltage of the data analyser is 12V, and the working current is 5A. To eliminate the interference of signals from other devices, the whole shell of the device is made of plastic. The biggest advantage of the data analyser is that the voltage can be changed from 12 V to 5 V according to the actual working environment. The analysis results of news data output by the data analyser will be encrypted in Lan format to cause data leakage and unnecessary troubles.

### Central controller

The central controller is an important device in the hardware area of the system. Its main job is to drive all the devices in the system and control the running state of each device. Once the system is in an abnormal state, the central processor will immediately conduct an internal analysis, quickly find the fault point and complete the problem control and solution. The central controller is located at the core of the system hardware area, and its connection with the surrounding devices is interactive. The main controller must be connected to all the devices inside the system. Because of the large amount of work, the central controller is connected to important devices, and all the connections are realized indirectly.

The interface between MP/MC and DCNF is used in the central controller. All the instructions output by the device are 64-bit data strips, and the device realizes the control function in signal wave analysis. To improve the operating efficiency and fault repair rate of the system, the central controller also needs to receive the information reading and writing requests of the other devices. The effective working voltage of the central controller is 3 V∼6 V, and the conversion circuit and control circuit are integrated into the controller, which is controlled by static Flash to avoid the automatic tampering of the internal information of the circuit, simplify the circuit connection complexity and improve the operation efficiency.

### Sensor design

**Figure 4:**
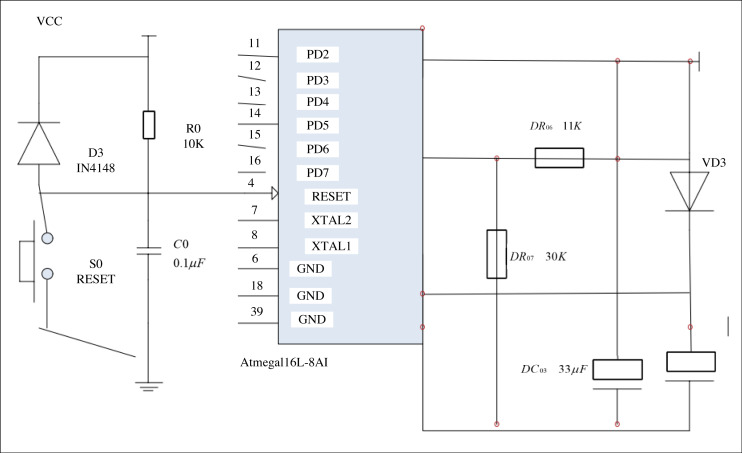
Internal structure diagram of sensor.

The function of the sensor in the hardware area of the system is to connect the sending signals of all devices in the system and complete the integration of data transmission characteristics. The sensor’s sensitivity is 68D, and the maximum output frame rate is 35 Hz. The external interface types of the sensor are the LAN interface, USB interface, 3IO interface and RS232 interface, which ensure the application range of the sensor. The internal structure diagram of the sensor is shown in Fig. 4.

 The internal structure of the sensor is an all-solid structure, which can reduce external interference waves and improve the transmission efficiency of the sensor. The sensor supports N/S two-stage conversion detection; its running protocol is a CAN2.0 communication protocol. The maximum current consumption parameter of the device is 80ma; the response start-up speed is 5ms, the effective ambient temperature range is −40 °C∼+ 40 °C, and the output impedance is less than 500. It can reduce the load data output by the sensor, improve the running speed and protect the health of the battery in the system. The dielectric strength of the sensor is less than 100 uA, and the independent linearity of the device is 0.05%, which reduces the bit error rate of news data transmission.

## Network Transmission Protocol based on an AI Algorithm

AI technology simulates and expands human behavior, widely used in many fields. The essence of an AI algorithm is to integrate various devices to give it thoughts and behaviors about people so that it can respond similarly to human behavior when it is out of the control of the human brain (Şahap Atik, 2022). Therefore, this article adopts the network transmission protocol based on an AI algorithm to improve the depth and security of news data transmission feature extraction.

The network transmission protocol based on an AI algorithm has the normal network communication protocol function and can encrypt the transmitted news data text. Meanwhile, it improves the running efficiency and facilitates the subsequent feature extraction and deep mining of news data. The essence of this transmission protocol is to process the normal network protocol in two dimensions by using a discrete Fourier rule algorithm and then realize the activation and docking of the special attributes of the protocol. Discrete Fourier Transform (DFT) is the discrete form of Fourier transform in time domain and frequency domain, which transforms the time domain sample of a signal into its DTFT frequency domain sample. Formally, the sequences at both ends of the transform (in time domain and frequency domain) are of finite length, but in fact, both sets of sequences should be considered as the principal sequences of discrete periodic signals. Even if DFT is applied to a discrete signal of finite length, it should be regarded as the transformation of its periodic continuation. In practice, fast Fourier transform is usually used to calculate DFT, whose core can be formulated as:

(1) \begin{eqnarray*}\widetilde {X} \left( k \right) =\sum _{n=0}^{N-1}\widetilde {x} \left( n \right) {W}_{N}^{kn},0\lt k\lt N-1\end{eqnarray*}

(2) \begin{eqnarray*}\widetilde {x} \left( n \right) = \frac{1}{N} \sum _{n=0}^{N-1}\widetilde {X} \left( k \right) {W}_{N}^{-kn},0\lt n\lt N-1.\end{eqnarray*}

According to the data characteristics to be analyzed and processed, it is connected to the system so that the system data carrier is synchronized with the random code. The frequency domain of the network transmission protocol will fluctuate accordingly. The fitting function calculation formula of fluctuation amplitude and the docking attribute of the network transmission protocol is as follows:

(3) \begin{eqnarray*}G \left( X \right) \sum _{r=1}^{n}x \left( r \right) \ast \frac{y \left( r-n \right) }{\mathrm{\ell }} +\int \nolimits \nolimits _{r=1}^{n}y \left( n \right) /\rho .\end{eqnarray*}

Among them, *n* represents the measurement relation index of current network transmission protocol signal data; *r* represents the executable domain of the current news data transmission signal; ℓ represents the measured relation value of the frequency domain of the currently extracted news data propagation characteristics; $x \left( r \right) $ represents a data sequence representing the control end of news data dissemination characteristics; $y \left( n \right) $ represents the news data transmission signal receiving sequence representing the central control unit of the system; *ρ* represents the blocking value of signal transcoding in the process of activating special attributes of network transmission protocol. According to the above calculation, the reasonable allocation of news data dissemination characteristics can be realized.

**Figure 5:**
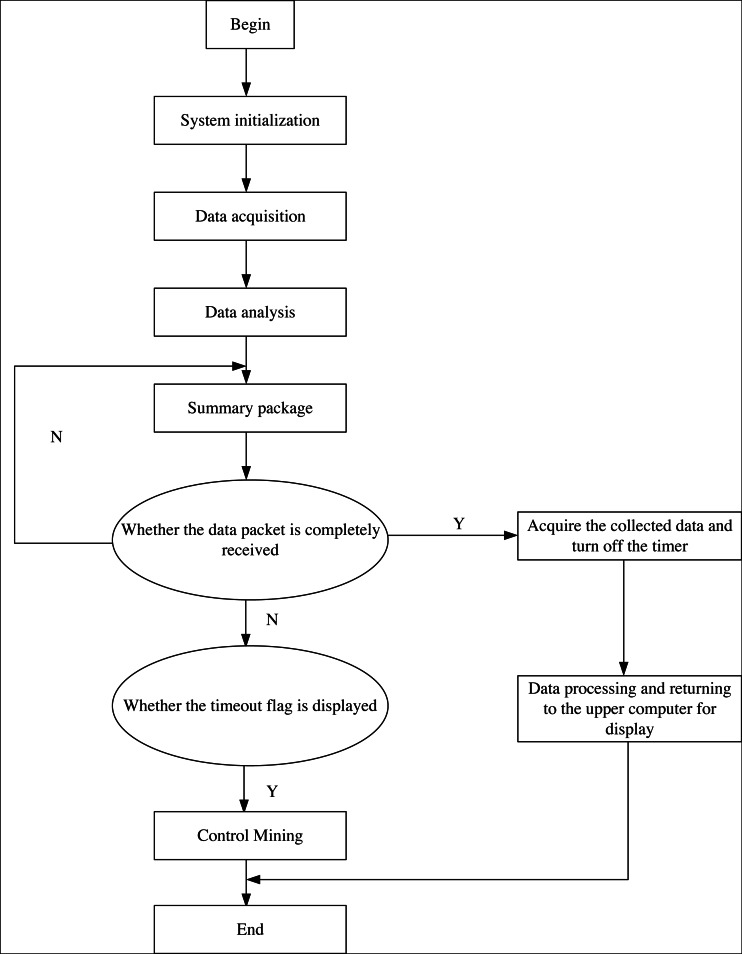
The operation process of the system.

The specific implementation process of the system software is shown in Fig. 5.

(1) Initialize the system. The central controller of the system drives all the hardware devices in the hardware area, the data collector collects the news data packets that need to be mined, denoises the data after collection, and finally transmits the data vector set with a completed format to the data analyzer.

(2) The collaboration software area of the data analyzer builds a characteristic communication model, extracts the communication characteristics of news data and the communication orientation of big news data, and finally summarizes such information into a data packet, which is stored in the system database.

(3) Send the data packet to each data node associated with the network transmission protocol, calculate the news data propagation characteristics, and finally send the calculation results to the system’s central controller to realize the mining of news data propagation characteristics.

## Experiment and Analysis

### Experimental environment and data set

Our experiments are conducted on an i5-13400F, Rtx 3080 device, and the network model is implemented under the Caffe framework and Matlab software. The total number of rounds of the experiment is 30. Meanwhile, we note the batch size to 64 and the initial learning rate to 0.004. to train the model, we adopt Adam as the optimizer and employ the momentum of 0.95. Besides, the decay of the weight is 0.0001. We have applied the Microsoft News Dataset to conduct our experiments. The acquired data consists of three views, namely text (word granularity), picture (tag granularity) and video (tag granularity). The news data are all from the news sections of entertainment, sports, military and science and technology. Selecting news data in the same period as experimental data can effectively reflect the authenticity of news data. The average accuracy and real-time mining are selected as evaluation indexes.

### Selection of the characteristic keywords

**Figure 6:**
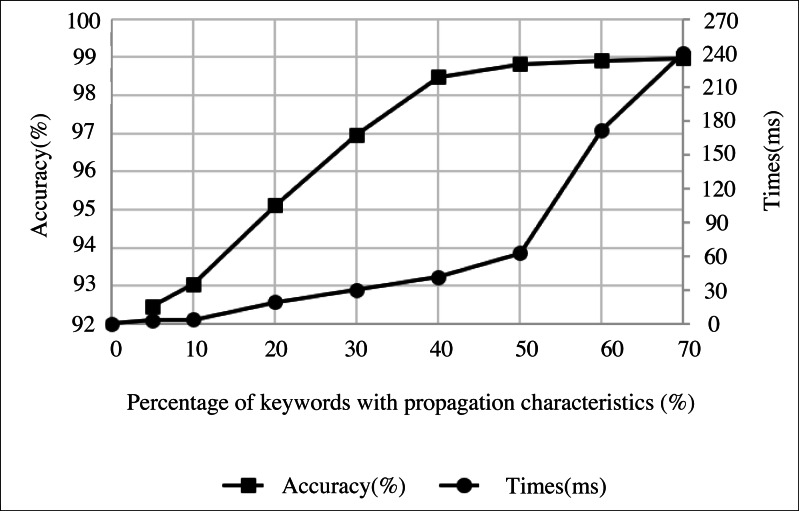
The influence of the number of communication characteristic keywords on the mining accuracy.

In news data dissemination characteristics and mining system operation, selecting keywords of dissemination characteristics directly affect the effectiveness of the system for news communication. A few communication characteristic keywords can’t accurately express the news theme, resulting in a poor mining effect. However, an excessive number of communication characteristic keywords will also negatively impact the experiment. Therefore, before mining news data, we must first calculate the number of communication characteristic keywords with the highest weight value that need to be sent into the system. Therefore, this article comprehensively considers the = value from mining accuracy and mining time. Figure 6 shows the influence of the number of communication characteristic keywords on the mining accuracy.

 When the selected number of communication characteristic keywords accounts for about 40% of the total number, the growth rate of mining accuracy begins to slow down. When it accounts for 50%, the mining time begins to increase sharply. Therefore, the median value of 45% can be selected as the proportion of the number of communication characteristic keywords.

### Comparison of different methods

To verify the mining performance of this system on large-scale news data, the AI algorithm in this system is compared with the blockchain algorithm (Stachowski, Fiebig & Rauber, 2020) and data analysis algorithm (Xiao, Zhou & Zhao, 2020).

**Figure 7:**
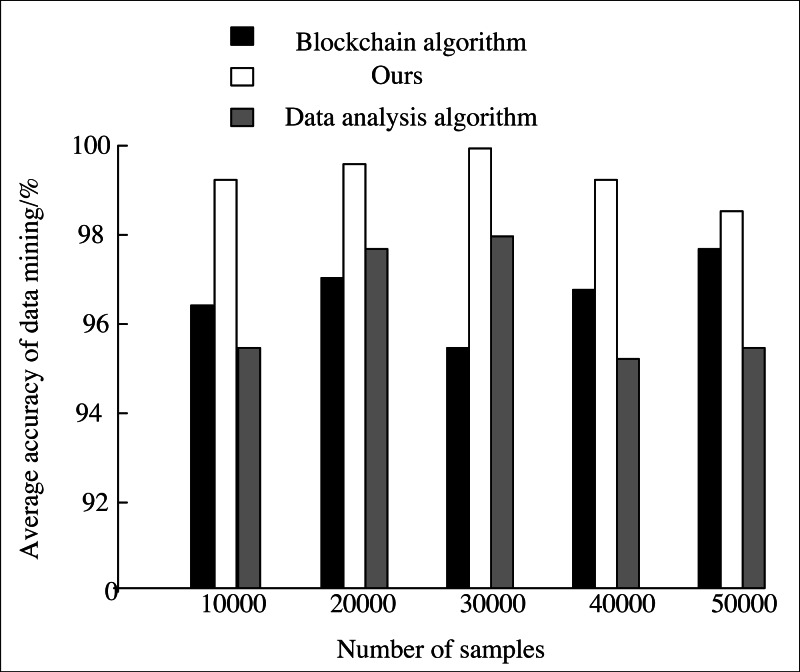
Comparison of average accuracy.

**Figure 8:**
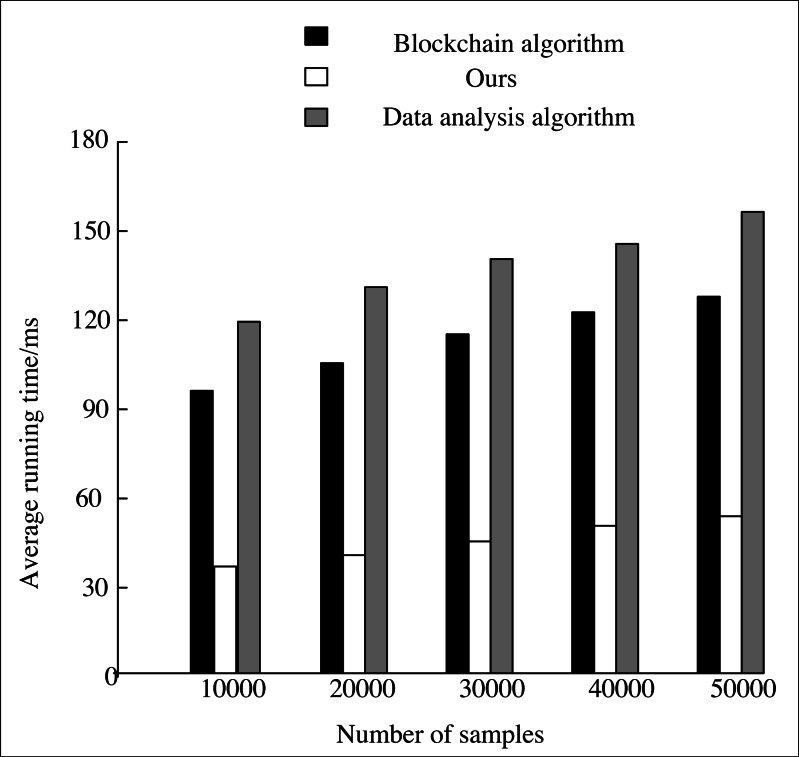
Comparison of average running time.

The mining performance and running time of news data with scales of 10,000, 20,000, 30,000, 40,000 and 5,000 are compared, respectively. The accuracy and the running time of mining data with three algorithms in different sample numbers are counted. The experiment is repeated ten times, and the average value is calculated. The statistical results are shown in Figs. 7 and 8.

From the experimental results in Fig. 7, when using our method to process large-scale news data, it still has a high mining accuracy when there are many samples. The average mining accuracy is higher than 98% when there are different samples. The average mining accuracy is higher than the other two algorithms, which effectively verifies that this algorithm has a high mining accuracy for news data.

From the experimental results in Fig. 8 that the average running time of the three methods for mining news data increases with more samples. The average running time of our algorithm for mining news data with different sample numbers is all lower than 60 ms, which is lower than that of the other two algorithms. At the same time, the other two algorithms are all higher than 90 ms, which shows that the proposed algorithm has high mining execution efficiency and convergence speed.

**Figure 9:**
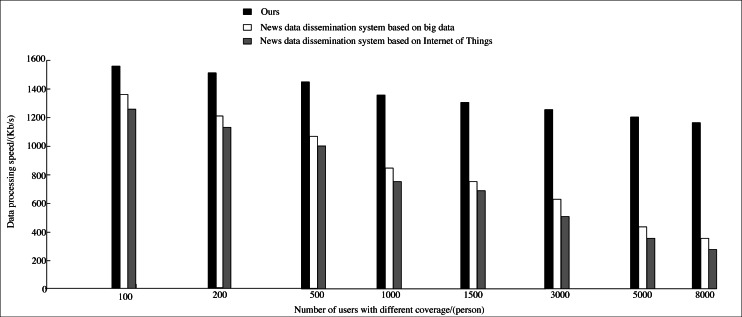
Comparison of different systems.

In addition, the news data processing speed of this system is compared with the news data dissemination system based on big data and IoT news data dissemination system (Hao, Liu & Wang, 2022). The results are shown in Fig. 9.

When the number of users covered is 100, there is no obvious difference in the processing speed of news data among the three systems. When more users are covered, the data processing speed of the three systems decreases, but there are obvious differences. While the data processing speed of the news data dissemination system based on big data and IoT drops sharply. In this article, the IoT technology and AI algorithm are combined to realize the efficient processing of news data. However, the speed of data processing is not apparent. By comparison, it can be found that compared with other news data dissemination systems, the proposed method can process data faster and expand news communication to a certain extent.

## Conclusion

To obtain the efficient and accurate transmission of news data, we propose a feature mining system based on IoT and AI. By the designed data collector, data analyzer, central controller and sensors, we can call for the regional function of the system software. Besides, we embed AI-based network transport protocols, which can be implanted into the system software field and can further improve the operating efficiency of the system. Experiments can demonstrate that mining news data features can achieve an accuracy of over 98% and our dissemination system can cost less time to process data, which can effectively guide the upgrading of news communication mode and improve the efficiency of news communication.

##  Supplemental Information

10.7717/peerj-cs.1428/supp-1 Supplemental Information 1 Code

## Additional Information and Declarations

### Data Availability

The following information was supplied regarding data availability: The code is available in the Supplemental File. The data is available at Github: https://msnews.github.io/; Fangzhao Wu, Ying Qiao, Jiun-Hung Chen, Chuhan Wu, Tao Qi, Jianxun Lian, Danyang Liu, Xing Xie, Jianfeng Gao, Winnie Wu and Ming Zhou. MIND: A Large-scale Dataset for News Recommendation. ACL 2020.
